# High-glucose administration induces glucose intolerance in mice: a critical role of toll-like receptor 4

**DOI:** 10.3164/jcbn.18-81

**Published:** 2019-03-07

**Authors:** Xiandong Zhan, Lijuan Wang, Zhenhui Wang, Shiping Chai, Xiaobo Zhu, Weidong Ren, Xiaotong Chang

**Affiliations:** 1 Department of Endocrinology, the First Affiliated Hospital of North University of Hebei, Zhangjiakou, Hebei, 075000, China; 2 Institute of Pathogen Biology and Immunology, North University of Hebei, Zhangjiakou, No 11, Zuanshi South Road, Zhangjiakou, Hebei, 075000, China; 3 Department of Nuclear Medicine, the 251st Hospital of PLA, Zhangjiakou, Hebei, 075000, China; 4 Department of Medicine, North University of Hebei, Zhangjiakou, PR, 075000, China

**Keywords:** glucose, glucose intolerance, insulin resistance, inflammatory cytokines, toll-like receptor 4

## Abstract

Glucose converted from a diet has been considered a high-risk factor of type 2 diabetes mellitus (T2DM). However, it is not clear how it increases the risk of T2DM. Here, we investigated the effect of high-glucose administration on glucose tolerence in wild-type and toll-like receptor 4 (TLR4) knockout mice. Mice were intragastrically administered with high-glucose. The level of fasting blood glucose, insulin and intraperitoneal glucose tolerance were measured, and insulinogenic index and HOMA-IR were calculated at 1 week. To understand mechanism of glucose action, we also assessed blood glucose, glucagon-like peptide-1 and inflammatory cytokines levels at different time windows following high-glucose load. Our results show that 20 g/kg glucose load leads to glucose tolerance impairment and insulin resistance in wild-type mice. Following 20 g/kg glucose load, the levels of plasma interlukin-6 (IL-6) and tumor necrosis factor-α (TNF-α) increased significantly in wild-type mice, but not in TLR4 knockout mice. Moreover, 20 g/kg glucose load also impaired glucose-induced GLP-1 secretion in wild-type and TLR4 knockout mice. Our results indicate that high-glucose load leads to glucose intolerance with insulin resistance through impairment of GLP-1 secretion, increase of blood glucose levels via activating TLR4 and increasing levels of IL-6 and TNF-α in mice.

## Introduction

The prevalence of type 2 diabetes mellitus (T2DM) has been increasing sharply, resulting from both a complex inheritance and environment interaction and a wide variety of lifestyle factors, such as sedentary lifestyle, obesity, smoking, alcohol consumption, etc.^(1)^ Dietary factors are considered to play an important role in the development of T2DM because they can influence the digestion rate, metabolism and blood glucose response.^(2,3)^ Saturated fat intake is closely associated with insulin resistance and T2DM, which has been investigated extensively. However, the role of carbohydrate in the process is less clear.

Over the last decades, more evidence supported the correlation between digestible carbohydrate consumption and T2DM incidence,^(4,5)^ and was found that T2DM incidence is associated with the amount and types of dietary carbohydrates.^(5–7)^ Some studies indicate that the role of carbohydrate-rich diets in T2DM and other chronic diseases depends on the conversion of carbohydrate into glucose *in vivo*. A diet that produces higher blood glucose would increase the risk of T2DM,^(8,9)^ and even glycemic index (the area under the glycemic curve during a 2 h after consumption of 50 g of carbohydrate) was introduced to assess a potential role of diets in the development of T2DM.^(8)^ Two main mechanisms also have been hypothesized, one is the increase in insulin resistance, and the other is pancreatic exhaustion resulting from the increased demand of insulin.^(8)^ However, it is not clear how glucose as dietary source increases the risk of T2DM.

It has been reported that high sugar intake or glycemic index of the diet is associated with inflammation in mice and humans.^(10,11)^ The role of inflammation in T2DM development has been widely studied, and it is associated with the pathogenesis of T2DM.^(12)^ Inflammatory cytokine production is mediated through toll-like receptors (TLRs). TLRs is a major class of pattern-recognition receptors in mammalian cells. TLRs can induce the expression of proinflammatory cytokines through interactions with conserved pathogen-associated molecular patterns and subsequently activate nuclear factor-κB (NF-κB) signaling pathway, and produce proinflammatory cytokines in mammals, including humans.^(13)^ Among the TLRs, TLR4 is strongly associated with T2DM, and plays a critical role in the pathogenesis of insulin resistance and T2DM.^(14–16)^

Therefore, we hypothesize that an increase in T2DM incidence by dietary glucose is associated with TLR4 function. In this study, we investigated the effect of high-glucose administration on some parameters related to T2DM in wild-type and TLR4 knockout mice to understand the reason that dietary glucose increases T2DM incidence.

## Materials and Methods

### Animal and protocols

C57BL/6 wild-type and TLR4 knockout male mice from a homogeneous C57BL/6 background (the Department of Laboratory Animal Science, Peking University Medical College, Peking, China), weighing 20–22 g, were housed at a constant room temperature (22–25°C), humidity (45–55%), with a 12:12 h light–dark cycle and fed with a standard rodent diet and water *ad libitum* in the animal center of Hebei North University. All procedures involving in animals were approved by the Animal Utilization Committee of Hebei North University according to the Guidelines for Animal Care of Hebei North University. All efforts were made to minimize animal suffering and to reduce the number of animals used.

After a few days of acclimatization, normal C57BL/6 mice were randomly divided into three groups. Two wild groups (*n* = 8 in each group) were intragastrically administered with glucose at the dose of 10 g/kg (25% d-glucose) and 20 g/kg (50% d-glucose), the control group was intragastrically administered with physiological saline solution, respectively, after 12 h of starvation. Blood samples were collected into drof tubes from the retro-orbital sinuses for the subsequent measurement of glucose, inflammatory cytokines, glucagon-like peptide 1 (GLP-1) (the drof tubes containing dipeptidyl peptidase-4 inhibitor, 2 µl, 1 mM diprotin A; Sigma-Aldrich, St. Louis, MO), at different time points. At 1 week of treatment of oral high-glucose administration, intraperitoneal glucose tolerance test, and subsequent analysis of β-cell function and the homeostasis model assessment of insulin resistance were performed in various groups. Next, two TLR4 knockout mice groups were intragastrically administered with glucose of 20 g/kg (50% d-glucose) and physiological saline 0.9% solution, respectively, after 12 h of starvation. Subsequent blood collection and parameter measurement are identical to that used for wild-type C57BL/6 mice.

### Intraperitoneal glucose tolerance test (IPGTT)

Mice were intraperitoneally injected with glucose solution at dose of 2 g/kg. We collected 100 µl blood samples at 0, 15, 30, 60 and 120 min from the retro-orbital sinuses. The samples were centrifuged, and plasma was separated and stored at –80°C until the assay for blood glucose and insulin levels. The area under the curve (AUC) is calculated by the trapezoid method. IPGTT was widely used to evaluate insulin response to parenteral glucose load.^(17)^ In our study, we used IPGTT to evaluate insulin secretion capacity in mice. The levels of glucose and insulin at the 15 min time point were adapted to calculate insulinogenic index (IGI) (i.e., an index of β-cell function) with the formula: [15 min-insulin (µU/ml) – fasting insulin] / [15 min-glucose (mM) – fasting glucose].

The homeostasis model assessment of insulin resistance (HOMA-IR) was determined using the following a formula: {HOMA-IR = [fasting plasma glucose (mM) × fasting plasma insulin (µUI/ml)]/22.5}.^(18)^

### Laboratory analysis

Blood glucose concentrations were measured with a glucometer (Elite; Bayer Inc., Buenos Aires, Argentina). Insulin levels in plasma were determined by enzyme-linked immunosorbent assay kit (Epitope Diagnostics, Inc., San Diego, CA) in accordance with manufacturer’s protocols. Total GLP-1 was measured using a commercially available enzyme-linked immunosorbent assay kit (Epitope Diagnostics, Inc.) according to the instructions from the manufacturer. The interleukin-6 (IL-6) and tumor necrosis factor-α (TNF-α) levels in plasma were determined with an enzyme-linked immunosorbent assay kit (Bioscience, San Diego, CA) according to the manufacturer’s instructions.

### Statistical analysis

All results were expressed as mean ± SEM of values obtained from experiments. Statistical analysis was performed by one-way analysis of variance (ANOVA). The statistical significances of the differences between groups were further established by the Bonferroni/Dunn multiple comparison test. *P* values less than 0.05 were considered to be statistically significant.

## Results

### High-glucose load impairs glucose tolerance in wild-type mice

**Fig. 1:**
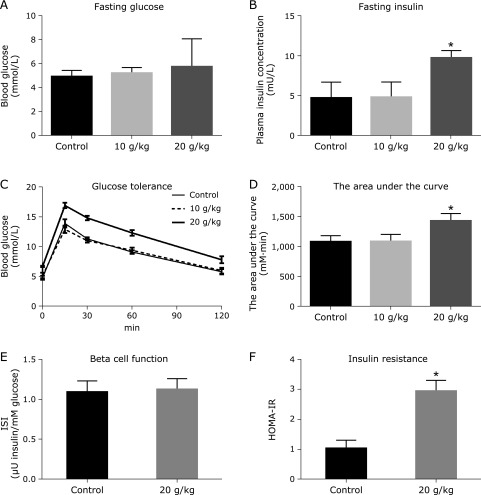
High-glucose load impairs glucose tolerance in wild-type mice. Wild-type mice (*n* = 8) were administrated with high-glucose (10 and 20 mg/kg, respectively) or saline 0.9% solution. At 1 week, the blood was collected for analyzing the fasting blood glucose (A), plasma insulin levels (B), glucose tolerance curve (C), the area under the curve (D), insulinogenic index (ISI) of islet β-cell (E), and the homeostasis model assessment of insulin resistance (HOMA-IR) (F). Differences between the treatment group and the control group are presented in the figure (*p<0.05). The values are mean ± SEM.

We first administered two different doses of high-glucose, 10 g/kg and 20 g/kg, to wild-type mice to examine their effect on blood glucose, plasma insulin levels, and glucose tolerance test. At 1 week of high-dose glucose administration, the mice displayed no significant change of fasting glucose concentration (Fig. 1A), but plasma insulin levels increased significantly (Fig. 1B) (*p* = 0.018), compared with control. The area under the curve (AUC) of glucose tolerance shows statistically significant difference between 20 g/kg-treated mice group and control (*p* = 0.023) (Fig. 1C and D). However, 10 g/kg glucose intake has no effect on these parameters.

We next analyzed insulin secretion and insulin receptor sensitivity in mice treated with 20 g/kg glucose load. We used IPGTT to evaluate insulin secretion capacity. The levels of glucose and insulin at the 15 min time point were adapted to calculate insulinogenic index (IGI). No statistically significant difference for IGI was detected between treated mice and control (Fig. 1E). MOHA-IR was used to evaluate insulin sensitivity. The MOHA-IR of mice treated with glucose (20 g/kg) is statistically significant different from that of the control (Fig. 1F) (*p* = 0.016). These results indicated that high-glucose load (20 g/kg dosage) can lead to glucose tolerance impairment through causing insulin resistance in wild-type mice.

### The alternation of the levels of blood glucose, pro-inflammatory cytokines and GLP-1 following high-glucose load in wild-type mice

**Fig. 2:**
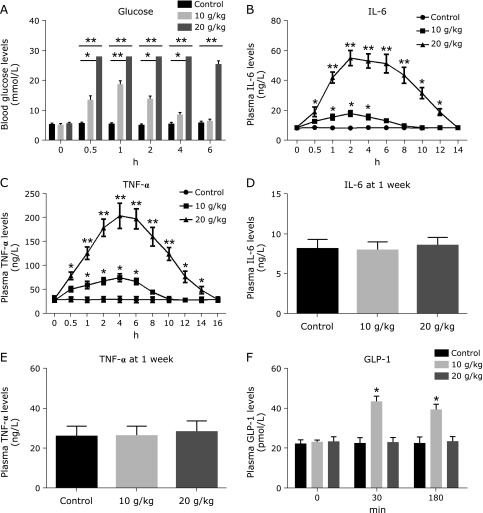
The changes of the levels of blood glucose, pro-inflammatory cytokines and GLP-1 following high-glucose load. Wild-type mice (*n* = 8) were administrated with high-glucose (10 and 20 mg/kg, respectively) or saline 0.9% solution, and blood were collected at different time windows for analyzing blood glucose concentrations at 0–6 h (A), interleukin-6 (IL-6) levels at 0–14 h time point (B), tumor necrosis factor-α (TNF-α) levels at 0–16 h time point (C), and IL-6 (D) and TNF-α levels (F) at 1 week after high glucose administration, as well as GLP-1 levels (F) at 30 and 180 min following high glucose load. Differences between the treatment group and control group are presented in the figures (*p<0.05, **p<0.01). The values are mean ± SEM.

The effect of high-glucose administration is distinctly associated with the translocation of glucose into the systemic circulation. Therefore, we measured blood glucose concentration in wild-type mice at several time points following high-glucose intake. Blood glucose concentration increased sharply and was higher than 27.8 mM at 30 min and lasted for 4 h in mice treated with 20 g/kg glucose, which is statistically different from that in the control group at each time point (Fig. 2A) (*p* = 0.002). Due to the association of high sugar intake with inflammation in mice and humans,^(10,11)^ and the important role of inflammatory cytokines in insulin resistance,^(19,20)^ in the meantime, we also analyzed plasma levels of IL-6 and TNF-α. The plasma level of IL-6 rose significantly from the 0.5 h time point and peaked at the 2 h time point in mice treated with 20 g/kg glucose, and a statistically significant difference was observed when compared to that in the control mice (Fig. 2B) (*p* = 0.003). However, the plasma TNF-α levels elevated significantly starting from the 0.5 h mark and peaked at the 4 h mark in mice treated with 20 g/kg glucose, and a statistically significant difference was observed when compared to that in the control mice (Fig. 2C) (*p* = 0.002). The increase in these pro-inflammatory cytokines persist 14 and 16 h, respectively, following high glucose load. At 1 week after high glucose load, these cytokines did not elevate, and there were not significant differences between high glucose treated and control mice (Fig. 2D and E).

Moreover, it has been reported that GLP-1 increases glucose-induced insulin secretion and has been used for glycaemic control and anti-inflammation.^(21,22)^ Oral administration of glucose results in two overlapping phases of GLP-1 secretion at 15–30 min and 1–3 h.^(23)^ Therefore, we analyzed plasma GLP-1 levels at 30 min and 180 min after high-glucose load. Glucose intake (20 g/kg) blunted GLP-1 secretion, and plasma GLP-1 levels did not significantly change, but glucose load at 10 g/kg increased significantly plasma GLP-1 level (Fig. 2F). Together, these results suggest that high-glucose effect is associated with significant increase in blood glucose, inflammatory cytokine IL-6 and TNF-α, and the impairment of GLP-1 secretion.

### TLR4 knockout prevents glucose intolerance induced by high-glucose load

**Fig. 3:**
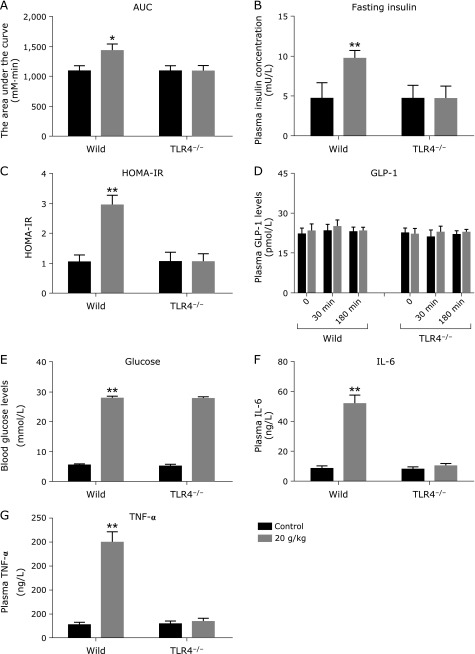
TLR4 knockout prevents glucose intolerance induced by high-glucose. TLR4 knockout mice (*n* = 8) were administrated with 20 g/kg glucose or saline 0.9% solution. At 1 week, the AUG of glucose tolerance (A), fasting insulin (B) and HOMA-IR (C) were analyzed. To understand the mechanism, blood was collected at 30 and 180 min following high-glucose administration for measuring plasma GLP-1 (D), and at the 4 h time point following high-glucose load for measuring blood glucose (E), plasma levels of interleukin-6 (F) and tumor necrosis factor-α (G). Differences between the treatment group and the control group are presented in the figures (*p<0.05, **p<0.01). Values are mean ± SEM.

We further examined if TLR4 receptor is required for glucose intolerance induced by high-glucose load. TLR4 knockout mice were intragastrically administered with 20 g/kg glucose. At 1 week, TLR4 knockout mice displayed no significant difference in the AUG of glucose tolerance (Fig. 3A), fasting insulin (Fig. 3B) and HOMA-IR (Fig. 3C), compared with the control mice. To understand the mechanism, we collected blood at 30 and 180 min following high-glucose load for measuring plasma GLP-1, and at the 4 h time point for measuring blood glucose, plasma levels of IL-6 and TNF-α. Plasma GLP-1 at 30 and 180 min did not respond to high-glucose load (Fig. 3D). Although their blood glucose concentrations were significantly increased (Fig. 3E), plasma levels of plasma IL-6 and TNF-α did not rise at the 4 h time point following high-glucose load (20 g/kg) (Fig. 3F and G). These results suggest that TLR4 is essential for glucose intolerance induced by high-glucose load, or TLR4 mediated the glucose intolerance.

## Discussion

In this study, we demonstrated for the first time in C57BL/6 mice that high-glucose administration can result in glucose intolerance. The glucose intolerance by glucose load is due to insulin resistance, as verified by MOHA-IR analysis. Particularly, we found that single intragastric administration of high-dose of glucose can lead to glucose tolerance impairment, suggesting that the quantity of glucose administration is crucial, and has nothing to do with frequency of glucose administration.

Glucose tolerance refers to the ability of the human body to tolerate the oral glucose challenge, which requires an increase in insulin secretion from islet β cells and insulin-mediated elevation of glucose uptake by peripheral tissue. When an individual is unable to respond to the glucose load from a meal and increases postprandial blood glucose, glucose intolerance occurs. Glucose intolerance is considered to be a pre-diabetic state and a high-risk factor for developing T2DM with a rate of 5–10% each year.^(24)^ It is also a risk factor for cardiovascular disease and metabolic syndrome.^(2)^ Glucose intolerance caused by high-glucose load, as shown by our results, may be crucial reason that dietary glucose increases T2DM incidence, and other chronic diseases risk.

In this study, the dose of glucose that induces glucose intolerance in mice is 20 g/kg body weight, a far higher dose than that usually used. Following intragastric administration of glucose, blood glucose concentration sharply increased beyond 27.8 mM and lasted for 4 h. It is very likely that persistent higher blood glucose level plays a direct pathogenic role in the process of disease development. The role of high-level of blood glucose in the induction of insulin resistance has been extensively studied. Some potential mechanisms include the reduction of insulin receptor kinase activity, the degradation of insulin receptor substrate, and the generation of reactive oxygen species.^(25)^ Recent studies are focused on the effect of inflammation and the immune system on the pathophysiological processes of insulin resistance.^(12)^ IL-6 and TNF-α are the most important pro-inflammatory mediator that are strongly associated with the development of insulin resistance,^(26,27)^ It was reported that IL-6 can directly inhibit insulin receptor signal transduction, and result in insulin resistance in primary mouse hepatocytes and the human hepatocarcinoma HepG2,^(28)^ and that IL-6 depletion in obesity mice by injection of IL-6-neutralizing antibody selectively increases hepatic insulin sensitivity.^(29)^ TNF-α function deficiency through targeted mutations in TNF-α gene or both of its receptors can result in marked improvement of insulin sensitivity in dietary, chemical, or genetic models of rodent obesity.^(30–32)^ Therefore, we measured plasma IL-6 and TNF-α levels following high-glucose load in mice. We demonstrated that high glucose load significantly increased plasma IL-6 and TNF-α levels, and IL-6 peaked earlier than TNF-α. The increase in pro-inflammatory cytokines persists 14–16 h following high glucose load. Kumar’s study in cultured human leukemia Jurkat T cells showed that high glucose exist dose-response effects on pro-inflammatory cytokines production.^(33)^ Other evidences also have indicated that higher blood glucose leads to macrophage infiltration as well as overexpression of numerous inflammatory cytokines, including IL-6 and TNF-α in lymphocyte, liver and other tissues.^(34–36)^ Moreover, plasma levels of these cytokines at the time point of 1 week after high glucose administration did not elevate, and there were not significant difference with control mice. This result suggests that rapid increase in pro-inflammatory cytokines within 14–16 h following high glucose load is crucial to insulin resistance, and that the insulin resistance is irreversibility, at least in the period observed.

It has been shown that pro-inflammatory cytokines act in autocrine and paracrine-dependent manner to perpetuate insulin resistance by interfering with several key steps of the insulin-signaling pathway in peripheral tissues. These include the activation of the c-Jun N-terminal kinase and NK-κB pathways, the stimulation of the phosphorylation of insulin receptor substrate-1, the reduction of glucose transporter-4 in adipocytes.^(37,38)^ The elevated level of TNF-α can impair serine phosphorylation in adipocytes and peripheral tissues and reduce protein expression of glucose transporter 4, insulin receptor and insulin receptor substrate-1 (IRS-1).^(26)^ On the other hand, IL-6 can induce the expression of the suppressor of cytokine signaling protein 3 (SOCS-3), a potential inhibitor of insulin receptor and negatively regulate the phosphorylation of IRS-1.^(27)^ Moreover, inflammatory cytokines also induce islet β-cell dysfunction, and trigger β-cell apoptosis and subsequent insulin deficiency.^(37)^ However, in our study, insulinogenic index does not demonstrate abnormal pancreatic β-cell function in mice with glucose intolerance. This is perhaps associated with blood glucose concentration and time of duration. Yan *et al.*^(17)^ used the hyperglycemic clamp method in mice to show that continuously intravenous glucose load (blood glucose 16.7 mM) compromises first-phase insulin response and decreases insulin level in islet β cells. Moreover, the impairment becomes more severe over time with continuous glucose load.

Due to the fact that TLR4 is associated with insulin resistance and the production of inflammatory cytokines, including IL-6 and TNF-α,^(14–16)^ we examined the effect of high-glucose load on glucose tolerance in TLR4 knockout mice. Intriguingly, high-glucose load failed to impair glucose tolerance and did not change the levels of plasma IL-6 and TNF-α in TLR4 knockout mice, which suggests a critical role of TLR4 in increase of IL-6 and TNF-α levels and glucose intolerance and insulin resistance induced by high-glucose load.

Several lines of evidence using the cultured human monocyte cell lines have shown that high glucose dose- and time-dependently up-regulate TLR4 expression and increase cell surface receptor via PKC-α and PKC-δ, meanwhile, increase NF-κB activation and significant proinflammatory cytokine secretion.^(39,40)^ However, little is known about how high glucose acts on TLR4 in the monocytes. Dasu speculated that high glucose action on TLR4 is associated with dimerization of TLR4 because the dimerization was regarded to be a prerequisite for TLR4 activation.^(39)^ Moreover, Dasu’s study showed that significant increase in TLR4 expression occurred at 6 h of incubation with high glucose.^(39)^ In the present study, rapid increase in pro-inflammatory cytokines suggested that high glucose is more likely to increase TLR4 activation, rather than TLR4 expression.

Enteral ingestion of nutrition, specifically glucose, can induce GLP-secretion from intestinal endocrine L cells in small intestine.^(23)^ GLP-1 plays a major role in glycaemic control through increasing glucose-dependent insulin release from pancreatic β-cells and suppressing glucagon secretion.^(41)^ Interestingly, we found that oral high-glucose load at dose of 20 g/kg is unable to increase plasma GLP-1 level in wild-type and TLR4 knockout mice. Because of the important role of GLP-1 in glycaemic control, it is reasonable to assume that impaired GLP-1 secretion is responsible for persistent higher blood glucose following oral glucose administration. Moreover, this finding suggests that excess intake of carbohydrate-rich diets may relate to GLP-1 secretion impairment in T2DM, which has shown by several groups.^(42,43)^ However, GLP-1 secretion was compromised in TLR4 knockout mice, suggesting that GLP-1 is not crucial for insulin resistance. Although other two studies have reported impaired GLP-1 secretion caused by a moderate high-sucrose diet (38.5%) and high levels of non-esterified fatty acids,^(42,43)^ the mechanism of nutrient-mediated GLP-1 secretion impairment is still not known, and further investigations are required.

In summary, our results show that high glucose administration results in glucose intolerance with insulin resistance through impairment of GLP-1 secretion, elevated level of blood glucose, activation of TLR4 and subsequently increased levels of IL-6 and TNF-α in mice.
